# Complement System: An Immunotherapy Target in Colorectal Cancer

**DOI:** 10.3389/fimmu.2022.810993

**Published:** 2022-01-31

**Authors:** Iman M. Talaat, Noha Mousaad Elemam, Maha Saber-Ayad

**Affiliations:** ^1^ College of Medicine, University of Sharjah, Sharjah, United Arab Emirates; ^2^ Sharjah Institute for Medical Research, University of Sharjah, Sharjah, United Arab Emirates; ^3^ Faculty of Medicine, Alexandria University, Alexandria, Egypt; ^4^ Faculty of Medicine, Cairo University, Cairo, Egypt

**Keywords:** complement system, colorectal cancer, immunotherapy, tumor microenvironment, tumor immunity

## Abstract

Colorectal cancer (CRC) is the third most common malignant tumor and the second most fatal cancer worldwide. Several parts of the immune system contribute to fighting cancer including the innate complement system. The complement system is composed of several players, namely component molecules, regulators and receptors. In this review, we discuss the complement system activation in cancer specifically CRC and highlight the possible interactions between the complement system and the various TME components. Additionally, the role of the complement system in tumor immunity of CRC is reviewed. Hence, such work could provide a framework for researchers to further understand the role of the complement system in CRC and explore the potential therapies targeting complement activation in solid tumors such as CRC.

## Introduction to Colorectal Cancer

Colorectal cancer (CRC) is the third most common malignant tumor and the second most fatal cancer in the world. In 2018, 1.8 million new CRC cases were recorded, with 881,000 deaths, accounting for approximately 10% of all new cancer cases and deaths worldwide (1). By 2035, the number of new cases is expected to reach about 2.5 million (2). Twenty-five percent of the newly identified patients are diagnosed with metastatic illness, and 40% will develop metastases within a year (3).

Despite advancements in treatment modalities, patients with metastatic CRC (mCRC) have a 5-year survival rate of approximately 15% (4). Surgery, chemotherapy, and radiotherapy are the common conventional treatments for CRC and can be used in combination depending on the location and course of the disease (5, 6). Because of their non-specificity and cytotoxicity, several side effects have been reported (7). Additionally, about half of the patients suffer from recurrence despite neoadjuvant treatment (8). As a result, more effective and alternative treatments for CRC patients are fundamental.

The understanding of the genomic landscape of CRC, via sequencing techniques, has yielded important hints about the significant pathways and mechanisms underlying cancer formation. These data have led to the discovery of cutting-edge therapies based on specific genetic markers. Even with these improvements, survival rates for mCRC patients have remained dismal, with some genetic mutations, as RAS mutations, showing a significant role in restricting therapeutic choices. Other treatment approaches, like immunotherapy or anti-BRAF drugs, have only been shown to be beneficial in a tiny percentage of patients. Consequently, a better understanding of the molecular evolution of CRC is mandatory to pave the way for potential therapeutic options (4).

## Introduction to Immunotherapy in the Treatment of Colorectal Cancer

It has been established that infiltration of T cells into CRC improves the prognosis of the disease. T cells recognize self from non-self by the binding of T cell receptors (TCR) to major histocompatibility I (MHC I) that is expressed on the surface of tumor cells (9, 10). Furthermore, co-inhibitory molecules assist the tumor cells to escape the recognition and destruction by the immune system. Immune checkpoint inhibitors block those molecules on T cells, thus releasing the “brakes” of the cytotoxic T cells and enhancing their antitumor activity. Such immunotherapies include programmed cell death 1 (PD1) and cytotoxic T lymphocyte antigen 4 (CTLA4) inhibitors (11). After showing an initial success in the treatment of melanoma, immunotherapy has been evolving as a promising strategy for many solid tumors including CRC (12). A distinguishing feature of immunotherapy in contrast to other pharmacological anti-cancer therapies is its ability to exert a durable remission in selected patients on the long term (13), with an acceptable safety profile (14). Nivolumab and pembrolizumab have emerged as efficient PD1 antibodies to treat patients with metastatic deficient mismatch repair (dMMR) CRC (15). While cancer immunotherapy mainly involves the manipulation of the cytotoxic T-cell function or number, there are other immune factors that play a significant role in cancer treatment modalities. In view of the recognized role of the complement system in inflammation, some reports have recently advocated complement modulation as a potential immunotherapeutic tool in solid tumors, e.g. melanoma as well as ovarian (16), lung, breast, and colon cancers (17, 18).

## Introduction to the Complement System

The complement system is one of the first lines of defence against foreign pathogens or stressed cells, as a major part of our innate immune system. It is a network of soluble proteins, membranous receptors as well as regulators that can act in various tissues and are generated by the liver. The complement can be activated *via* various pathways including the classical, lectin and alternative (19, 20). The main action of the complement system is by inducing an immune reaction by activating the adaptive immune system and opsonizing pathogens, thus maintaining homeostasis. The classical pathway is initiated by the binding of the C1 component to IgG or IgM antibodies, forming antigen-antibody complexes (21). On the other hand, the lectin pathway (LP) is activated by the recognition of sugar residues such as mannan-binding-lectins (MBLs), collectins or ficolins, just like the C1 complex (22). Consequently, this could lead to the activation of the classical pathway C3 convertase (C4bC2a), an enzymatic complex that cleaves C3 component into C3a and C3b. The alternative pathway is activated by permissive surfaces and leads to the formation of the bioactive C3(H2O) and the alternative pathway C3 convertase: C3(H2O)Bb with the aid of factor D and factor B (23). These pathways would act synergistically to increase the level of the opsonin C3b at the target site (24). Consequently, this would trigger phagocytosis of the pathogen or stressed cells (23), via the formation of the C5 convertase and the complex with C6 and C7 that forms the membrane attack complex (MAC: composed of C5b, C6, C7, C8 and C9) (19). MAC would cause the formation of lytic pores, massive calcium influx, membrane permeabilization and cell death (25, 26). Once the complement system gets activated, opsonins are produced throughout the process including the components C3b, C4b and C1q, that bind to the target tagging it for phagocytosis by antigen-presenting cells (APCs), thus leading to its clearance. Other key mediators are anaphylatoxins that are released in the circulation in order to trigger inflammation. They activate macrophages, neutrophils, mast cells, basophils, and eosinophils, resulting in cytokine production leading to vasodilation, increase vascular permeability, and neutrophil extravasation and chemotaxis (27).

The complement system has a wide range of functions including orchestration of the immune-mediated clearance of apoptotic host cells and immune complexes. Besides, the complement cascade is also activated directly upon pathogen encounter. It is worth mentioning that the complement system modulates the activity of adaptive immune cells such as B and T cells. For instance, the activated complement C3 fragments could bind to the receptors CR1, CR3, and CR4, leading to macrophages activation and phagocytosis induction (20). In addition, C5a, a potent anaphylatoxin and an active fragment of C5, binds to C5a receptor (C5aR), and regulates macrophage polarization and activates the nuclear factor-κB (NF-κB) signaling pathway (28). This sheds the light on the potential role of such a pathway in various inflammatory disorders (29, 30).

**Figure 1:**
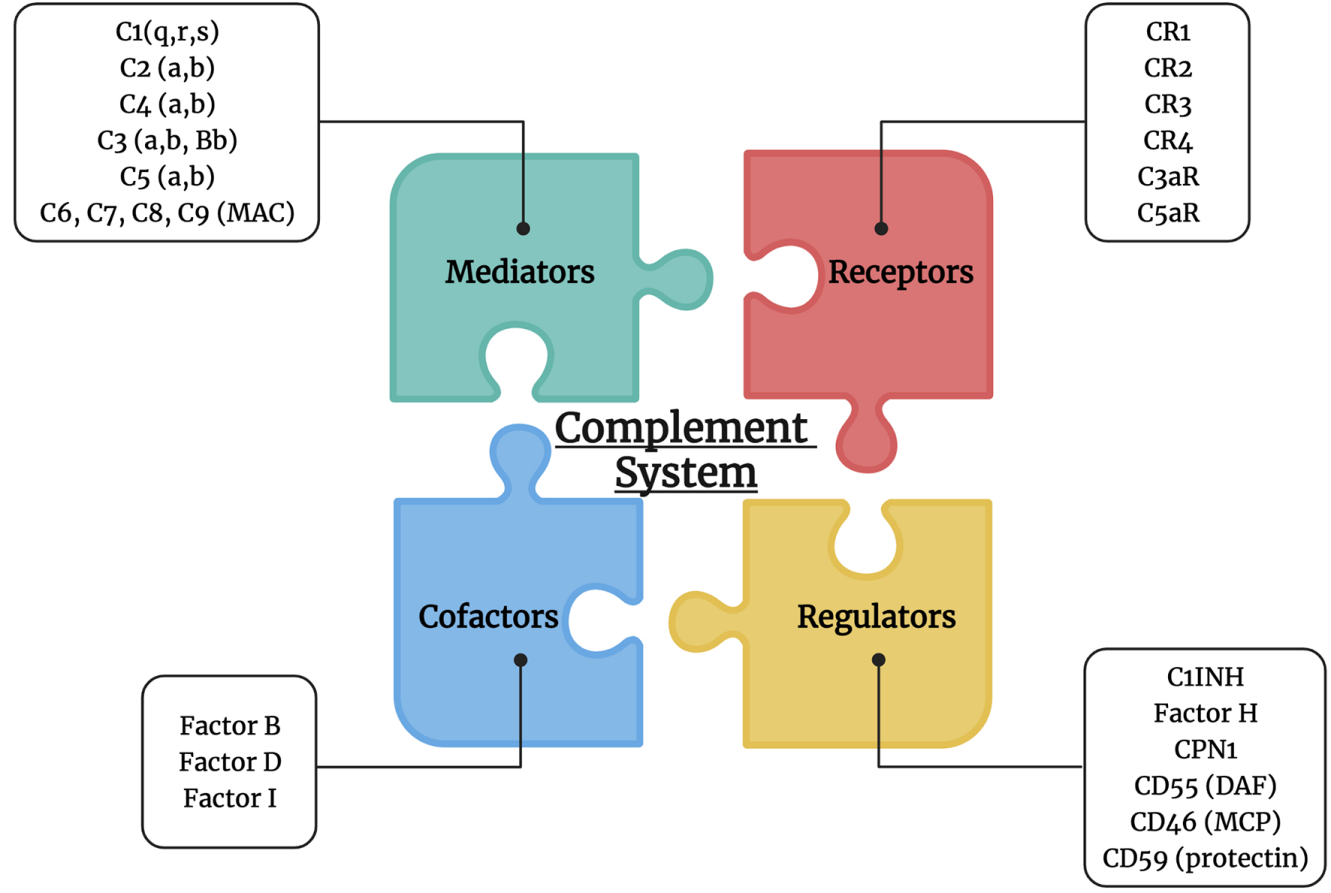
Complement system players. The complement system is composed of multiple mediators (C1, C2, C4, C3, C5, MAC: C6, C7, C8, and C9), cofactors (factor B, D and I), receptors (CR1, CR2, CR3, CR4, C3aR, and C5aR), and regulators (C1INH, factor H, CPN1, CD55, CD46, and CD59).

The function of the complement system is tightly controlled by multiple regulatory factors in order to protect the normal cells from unwanted casualties such as membrane-bound complement regulatory proteins (CRPs), which have a decay-accelerating activity and membrane cofactor activity (31). This includes the complement receptor type 1 (CR1/C4bp/CD35), factor H, membrane cofactor protein (CD46), decay-accelerating factor (CD55), and protectin (CD59) (**Figure 1**). CR1/CD35 is expressed by various immune cells such as neutrophils, eosinophils, monocytes, follicular dendritic cells, B and T lymphocytes (32). It acts as a cofactor in the cleavage of the C3b and C4b mediated by factor I, and it fastens the decay of the classical and alternative convertases (33). CD46 regulates T cell function by being a cofactor of factor I in C3b/C4b cleavage (34–36). On the other hand, CD55 which is expressed on several circulating blood cells, endothelial and epithelium cells, speeds up the decay of the classical and alternative C3 and C5 convertases (37, 38). Furthermore, CD59 inhibits the formation of MAC and the introduction of the C9 component into the lipid bilayer (39). Also, C1 inhibitor (C1INH) is known to inactivate the function of C1r, C1s, and MBL associated serine proteases (MASPs) (25). Another important regulator is carboxypeptidases, particularly carboxypeptidase N (CPN1), that inactivates the anaphylatoxin components C3a and C5a (40).

Any dysregulation (deficiency or overactivation) in the complement system can lead to various diseases involving the inflammation process and abnormal immune response, such as autoimmune diseases (41), and cancer (42). Like any other physiologic processes, complement system has regulators that aid in the maintenance of its function, that have been extensively reviewed before (24). In cancer, some of the complement components can be produced by the tumor and neighboring stromal cells. It remains unclear whether the tumor would benefit from the complement proteins or even develops possible ways of immune evasion.

## Role of Complement in the Tumor Microenvironment

The tumor microenvironment (TME) plays critical roles in carcinogenesis initiation and evolution, metastasis and relapse, as well as treatment resistance (43). Cancer cells, stromal cells such as immune cells and fibroblasts (44), in addition to the extracellular components make up the TME (45). Tissue-associated macrophages (TAMs), tumor-associated neutrophils (TANs), and myeloid-derived suppressor cells (MDSCs) are the immunosuppressive cell types that mostly infiltrate the TME (46). The proliferation and invasion of tumors have also been linked to dendritic cells (DCs) (47), cancer-associated fibroblasts (CAFs) (48), and regulatory T cells (Tregs) (49). Interactions between these cells and cancer cells are critical in tumor biological activity and response to treatment.

Interestingly, the immune microenvironment is rich in complement proteins and there is emerging evidence that complement components might have immunosuppressive functions in the TME by serving as a bridge between tumor-promoting and tumor-suppressing immune responses (50). Noteworthy, in malignant tumors, complement protein expression is found to be elevated. Since tumor cells and stromal cells both generate abnormal complement proteins, the TME’s complement system becomes aberrantly activated, promoting tumor development by curbing inflammation, stromal cell immunity, and tumor cell expansion, epithelial-mesenchymal transition (EMT), migration, and metastatic spread (51, 52).

In the TME, the major pathway implicated in complement activation is unknown. In patients with CRC, the LP was shown to be considerably higher than in normal individuals (53). Furthermore, complement proteins such as C1q and C5b-9 were found in colon, pancreatic, lung, and breast neoplasms as well as in melanomas (54, 55). Complement proteins were shown to drive and attract macrophages into cancer tissues, where IL-12 secretion by TAMs was suppressed by C5a (56). Additionally, in colon cancer liver secondaries, a tumor-inducing profile was acquired via the activation of NF-κB pathway. Consequently, C5a-mediated macrophage polarization with the expression of C5a receptor (C5aR) on TAMs was identified (28).

Previously, TANs have been linked to cancer progression, where it was found that complement system activation may lead to TANs chemotaxis within malignancies (57). This was further supported by the study by Dick et al. that reported C5aR to cause neutrophil dysfunction, while the study by Allendorf et al. discovered that C5a stimulates epithelial and endothelial cells to secrete leukotriene B4 (LTB4), which aids in neutrophil recruitment (58, 59). C5a, which is produced when complement is activated, promotes neutrophil recruitment by boosting the generation of the cytokine IL-1 (60). On the other hand, C5aR deficiency has been shown to prevent colon cancer tumor spread by lowering neutrophil infiltration in liver secondaries (61).

**Figure 2:**
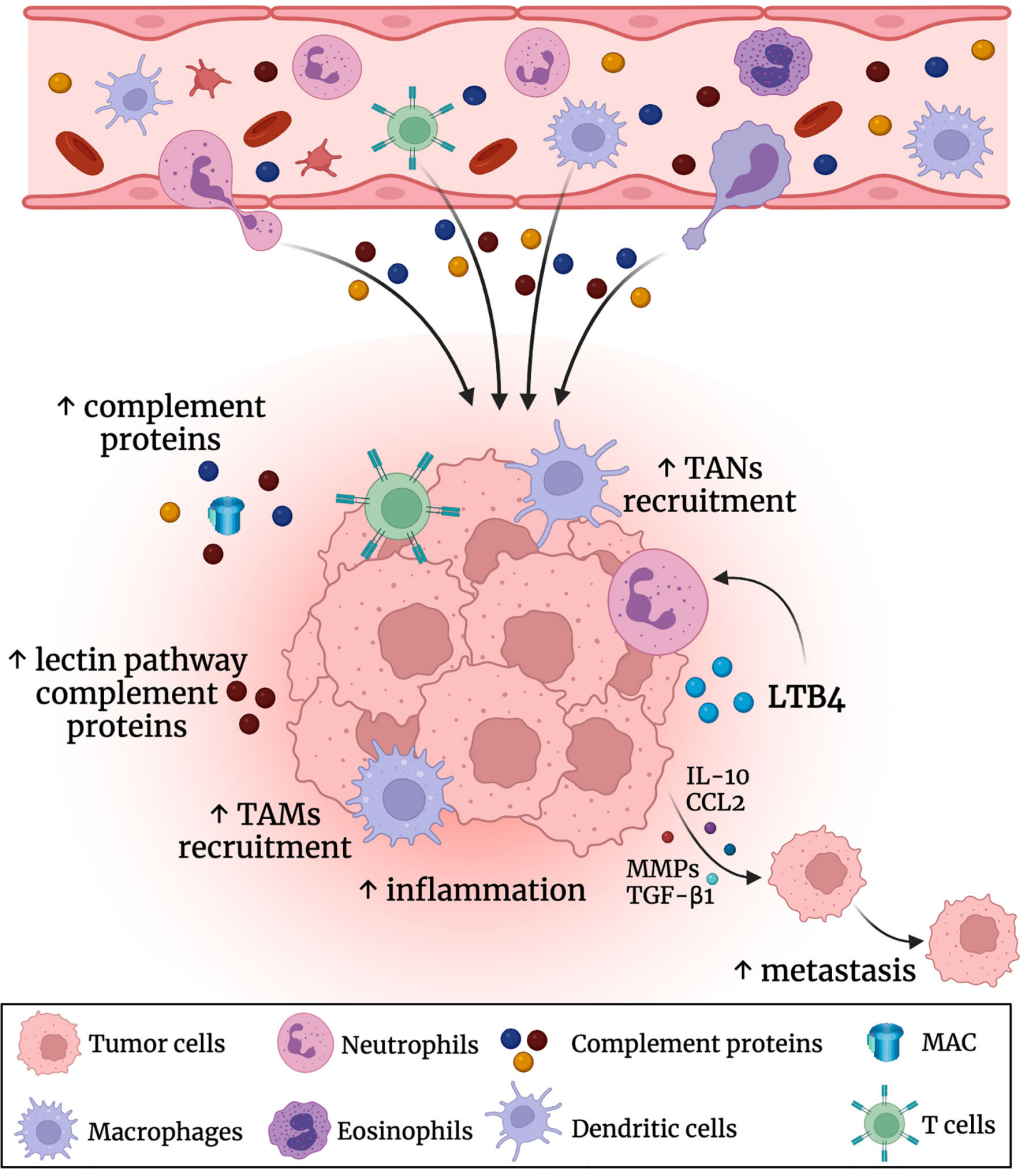
Role of the complement system in the CRC tumor microenvironment (TME). Complement proteins are produced by the liver into the circulatory bloodstream. There was an elevation in the levels of the complement proteins (especially the leptin pathway related proteins) in the colon TME. This promotes further inflammation and recruitment of tumor associated macrophages (TAMs) and tumor associated neutrophils (TANs). Also, the complement system triggers the secretion of IL-10, CCL2, TGF-β1 and metalloproteinases (MMPs) that could enhance CRC metastasis.

It was found that C3a-C3aR activation plays a key role in cell migration. Moreover, it was proved that inflammation and aberrant complement activation prompted metastasis in different cancers by TME status modulation, extracellular matrix (ECM) degradation, and tissue barriers disruption as well as enhancing the motility of neoplastic cells (62). In CRC, the activation of NF-κB pathway and the transcription factor AP-1 by the C5a–C5aR signaling may promote the production of matrix metalloproteinases MMP-1 and MMP-9, which were critical for the ECM breakdown (63, 64). Also, tumor cells produce C5a, which in turn increases the release of IL-10, transforming growth factor-beta (TGF-β1) and monocyte chemoattractant protein-1 (MCP-1), thus enhancing tumor metastasis (**Figure 2**) (61). Additionally, C3 and C4 may adhere to collagen and elastin in the arterial wall causing an enhancement of the vascular permeability, thus facilitating tumor spread (65, 66).

## Role of the Complement System in Tumor Immunity

The complement system was known to be an immune surveillance system against cancer due to its activity on tumor cells *via* MAC accumulation-mediated cell lysis or phagocytosis of opsonized cancer cells by macrophages and neutrophils. It is known that CRPs, whether soluble or membranous are elevated in cancer, with a differential expression across various cancer types (67, 68). In the tumor-immune interaction, complement-associated proteins play a vital role whether directly or indirectly by regulating tumorigenesis, development, and metastasis (69, 70). Like any other counterpart of the immune system, tumor cells manage to develop inhibitory mechanisms for the complement cascade in order to prevent complement-dependent cytotoxicity (CDC). However, several studies highlight the controversial role of the complement system in CRC, whether as a tumor suppressor or a tumor promoter (41). For example, mCRPs along with factors H and FHL-1 could be present in soluble forms that might attach to tumor cells, leading to tumor resistance to complement activation (71). Also, tumor cells may produce proteases that cleave complement molecules, and/or abolish MAC by endocytosis (72–74). This could induce multiple effects such as resistance to apoptosis and augmentation of complement resistance (75, 76). Moreover, several studies have shown that the complement activation may lead to chronic inflammation that results in the development of an immunosuppressive microenvironment and may even activate angiogenesis and cancer-promoting signaling pathways (77). For instance, mice deficient of the C3, C4, or C5aR components showed inhibition in their tumor growth in mice (78, 79). Also, the C5a component present in the TME could promote tumor cell growth by the recruitment of MDSCs and suppression of T cells (78, 80, 81). Also, the amount of C5a within the tumor was linked to the differentiation of regulatory T cells (82). Furthermore, the components C3 and C3a were reported to play crucial roles in cancer, where the disruption of C3a/C3aR axis caused a defect in the immune infiltration and leading to inhibition of tumor growth (83). Furthermore, C3a was found to promote T-cell apoptosis and MDSC recruitment, along with DCs and CD8+ T cells inhibition (83). Another possible mechanism of complement system through the C5a/C5aR pathway, where MDSCs upregulate the expression of programmed cell death 1 ligand (PD-L1) and repress the anti-tumor immune response (81, 84). Also, MDSCs induce the production and release of reactive oxygen and nitrogen species in order to suppress T cell function (81). On another note, CRPs were reported to be upregulated in various cancer types (85, 86), that promote the binding of C1q component to apoptotic cells, thus protecting tumor cells from lysis and inflammation (87). Therefore, blocking complement receptors in cancer could aid in enhancing the efficacy of the cellular immunotherapy (88, 89).

In CRC, tumor cells were found to produce C3 component thus leading to modulation of the response of macrophages and its anti-tumor immunity, via the C3a-C3aR axis and PI3Kγ signaling pathways (90). Also, another study reported that the C5a/C5aR1 axis could play a role in the tumor immunity and promote cancer progression (91). A previous study by Bao D. et al. showed that high levels of C3, CR4, and C5aR1 were associated with poor prognosis in CRC as well as immune infiltration levels of immune cells (92). Also, in colon carcinoma tissues, multiple complement elements including C2, C5, complement factor B (CFB), complement factor I (CFI), CR4, complement component 4 binding protein (C4BPB), CD46, CD55, and CPN1 were significantly higher than that in normal tissues. C1q was found to enhance tumor growth and to be highly expressed in CRC biopsies (55). Moreover, other studies found an increase in C3a serum concentration in colon cancer patients (93). Furthermore, the DAF/CD55 was selected to be a potential biomarker for poor prognosis in patients with colon cancer (92, 94), where tumors that express CD55 showed an increase in the CDC resistance (72, 95, 96). Also, LP components and serum levels of MBLs and MBL-MASP levels were increased in the serum of colon cancer patients and were reported to be a prognostic factor for recurrence and poor survival (53, 97). This highlights the value of the complement system in tumor immunoregulation, especially that of CRC.

Several studies claim that the chronic inflammatory state of the TME promotes neoplastic transformation (98). A recent study reported mutations in complement genes in CRC to be associated with the involvement of hypoxia gene expression as well as poor survival (99). In addition, there was an observed increase in the CD55 expression through the hypoxia inducible factor (HIF), leading to inhibition of CDC (99). In CRC, the C5a component was generated by serine proteases on the surface of tumor cells independent of complement activation (100), while the C5a/C5aR pathway was found to induce cell proliferation, motility, and invasiveness (101, 102). Blocking of this pathway was demonstrated to improve the response to PD-L1 therapy in CRC (103). On the contrary, another study described the tumor-derived C3a/C3aR signaling to affect TAMs by inducing the M2 phenotype and suppressing CD8+ T cells in CRC (90). Also, there was an improvement in response to PD-L1 therapy in C3-deficient tumors, thus suggesting that complement regulation of macrophages might affect T cell function and hence the therapeutic efficacy of PD-L1 antibodies (90).

## Therapeutic Aspects of the Complement System in CRC

Complement activation is a key driver of several immunological diseases, e.g., paroxysmal nocturnal hemoglobinuria (PNH), autoimmune hemolytic uremic syndrome and C3 glomerulopathy. Most clinical trials addressing the value of complement-related therapeutic targets are focusing on those diseases. As a result, the anti-C5 antibody, eculizumab, was approved by the FDA in the treatment of PNH in 2007. The other complement drug in clinical use is the C1 inhibitor, Cinryze, approved by the FDA for the treatment of hereditary angioedema as the first one of this class in 2010. Many other diseases are related to complement system derangement, including age-related macular degeneration, neuromyelitis optica and myasthenia gravis. Several clinical trials are ongoing to evaluate complement-related therapies in those diseases (104). Only a few complement-based lead molecules have been developed to therapies. A fewer number has gained the FDA or EMA approval. **Table 1** summarizes the basic features of medications acting on the complement system.

**Table 1:** Complement-targeting medications in different pathological diseases.

Specific Target (s)	Medication	Mechanism of Action	Modality	Current Clinical Use	Clinical Trial ID	References
C1, MASPs	Cinryze	Inhibition of lectin and classical pathways	Purified native protein	Hereditary angioedema	NCT02316353 NCT02584959	(105)
		Suppression of C1r/s and the MASPs activity		Under investigation in kidney transplant patients		
C3	AMY-101 (Cp40), compstatin derivative	Compstatin derivative with improved potency and safety profile compared to compstatin	Peptide	Acute respiratory distress syndrome due to COVID-19 (SAVE trial)	NCT04395456	(106, 107)
				Periodontal inflammation		
C3	Pegcetacoplan (APL-2)	Pegylated form of compstatin	Peptide	Paroxysmal nocturnal hemoglobinuria	NCT03500549	(108, 109)
					NCT04085601	
				Geographic atrophy	NCT02503332	
C5	Eculizumab	Inhibits the cleavage of C5 into C5a and C5b	Antibody	Paroxysmal nocturnal hemoglobinuria	NCT03500549	(108, 110–113)
				Acute hemolytic uremic syndrome	NCT01194973	
				Myasthenia gravis	NCT01892345	
				Neuromyelitis optica	NCT04355494	
				Severe COVID-19		
C5a Receptor	PMX53	C5aR1 inhibitors	Cyclic hexapeptide	Preclinical in mice	Preclinical	(114)
	PMX205			Suggestive for neurodegenerative diseases		
C5a Receptor	Avdoralib (IPH5401)	C5aR1 inhibitors	Monoclonal antibody	Bullous Pemphigoid	NCT04563923	(115)
				Advanced solid tumors	NCT03665129	
CD59	Anti-CD59	Inhibits MAC blocker “CD59”	Monoclonal or biphasic antibody	Multiple myeloma	Preclinical	(116, 117)
				Cervical cancer		
CD46	Anti-CD46	Inhibits CD46 and prevents C3b and C4b degradation by Factor H	Monoclonal or biphasic antibody	Cervical cancer	Preclinical	(116)

The development of high-resolution and dynamic-range analytical and structural methods, together with the introduction of complement-gene “knockout” models, formed the necessary foundations for a better understanding of the complement’s role in human pathologies including cancer (118). In cancer research, the therapeutic aspects of the complement system emerged as a consequence of unveiling its effect on TME components. Complement-related therapies may represent a promising strategy to overcome the failure of response to immunotherapy in different solid tumors including CRC (18).

Several concerns delayed the progress of complement-related therapeutics, as they are known to block an important arm of innate immunity. Hence, studies aimed to modulate rather than to completely block the complement receptors (119, 120). There are several disadvantages that appeared with the use of complement-based medications in the aforementioned autoimmune diseases. For example, complement inhibition impairs opsonization and bacteriolytic activity, thus increasing the risk of infections. This was reported in the case of eculizumab which was effective, however, associated with drawbacks, such as high risk of meningococcal infections and difficult pharmacokinetics. In order to overcome such challenges, the next generation complement-based medications were developed (104). These next generation high-potential drugs are rapidly progressing through clinical trials and are likely to change this field as they will have the potential of inhibiting the complement beyond C5 in various diseases and avoid the challenges associated with complement inhibition. Thus, next generation complement-based therapies have the advantage of owing a safer profile, in particular, a lower incidence of serious adverse effects compared to the older medications (104).

Interestingly, it was found that the effective doses used to treat cancer were much lower than that those used in the treatment of autoimmune diseases. One of the main therapeutic targets of complement system would be the C3 component as it is a point of convergence of the three complement pathways and a molecular hub for crosstalk with multiple pathogenic pathways. However, targeting C5 could inhibit the lytic effect of MAC, but leaves the complement build-up at the C3 level intact. Thus, it seems that C3 represents an attractive target for therapeutic modulation of the complement cascade. An example of a C3 inhibitor is compstatin which is a cyclin tridecapeptide that inhibits the cleavage of C3 to its active forms C3a and C3b. Compstatin and its newly developed analogues showed promising results in a wide spectrum of clinical applications (118).

In the last few years, a limited number of reviews and original studies have discussed the potential use of complement in therapeutics of different cancers (17, 18, 121, 122). Since the role of the complement system remains controversial, whether it is pro- or anti- tumorigenic, studies suggested suppressing the complement activation as a novel strategy for cancer treatment, probably using C5aR and C3aR blockers (25). Anti-complement agents in cancer treatment are considered a potential approach to be used in combination with traditional chemotherapies or immune checkpoint inhibitors without increasing myelosuppression; a well-known side effect of the chemotherapy (123). Also, complement inhibition has a promising role in enhancing the effect of immunotherapy, especially as the complement receptors C3aR and C5aR are expressed on CD8+ TILs and genetically engineered T cells (124). Additionally, targeting complement/C3aR/C5aR/IL-10 pathway has been suggested to synergize other treatment modalities, as it enhances the T-cell anti-tumor efficacy (124, 125). A previous study used fusion proteins (anti-PD-1-IL10) or (anti-CTLA4-IL-10), to be added for TILs expansion in the adoptive cellular therapy (124). Such a synergistic effect was further tested and confirmed in other studies on preclinical models of colon and lung cancers (103, 126), paving the way for future clinical trials. However, the risk and benefits of combining anti-complement therapies with other anti-cancer agents should be further investigated.

Several studies reported improving the complement-mediated monoclonal antibodies (mAbs) effects through genetic engineering, conjugation or even glycosylation. Others suggested turning a non-complement-fixing antibody into a complement-fixing antibody such as IgG1 and IgG3 that are most efficient in activating complement and CDC (127). For instance, the Fc part can be engineered to augment the CDC activity of therapeutic mAbs (128), while bispecific antibodies can be engineered to recruit complement effector functions (129), and alteration of the glycosylation was found to boost the lytic potential of mAbs (130).

Several studies scrutinized the therapeutic effect of blocking the complement system in mouse models. For instance, a study by Downs-Canner et al. demonstrated a reduction in tumor growth in a murine model of colon cancer, through different methods of complement depletion (using cobra venom factor) and inhibition (using *Staphylococcus aureus* superantigen-like protein 7) (131). Furthermore, an enhanced immune cell infiltration (namely CD8+ T cells) in the TME, as well as increased chemokines expression (CCL5, CXCL10, and CXCL11), were witnessed upon treatment of mice with these inhibitors (131). Since C5a/C5aR1 signaling axis is known to play a role in CRC TME immune infiltration, several studies explored the effect of complement C5 deficiency (especially *C5ar1*) where it was found to completely prevent CRC tumorigenesis. Also, this was accompanied by an increase in the levels of anti-inflammatory cytokines (IL-23, IL-9, IL-27, and IL-10) and suppression of the pro-inflammatory cytokines and chemokines (TNF-α, IL-1α/β, IL-6, IL-17A, IL-11, CCL2, CCL17, CXCL1, and CXCL5) (91). Moreover, the C5aR1 antagonist, PMX205, strongly impeded CRC growth, thus revealing the critical role of C5aR1 expression for colorectal tumorigenesis (91). Other C5aR antagonists, including PMX53, exerted efficient reduction in the tumor size and enhanced the effect of anti-cancer chemotherapy in mice (126, 132). It is worth mentioning that targeting the receptor C5aR rather than the components C3 or C5, allows opsonization to take place in order to protect cancer patients from the risk of acquiring bacterial infections. In addition, production of lytic MAC will be preserved upon inhibiting C5aR, hence favoring its anti-cancer effect. However, targeting C5aR leaves the other complement system components C3a uninhibited (132).

Several mAbs target tumor-specific antigens and are known to promote crucial anti-tumor activities. Moreover, these mAbs activate the immune system *via* the Fc portion through antibody-dependent cellular cytotoxicity (ADCC) and CDC (133). As previously mentioned, a successful complement activation could induce various immune responses against tumors (MAC formation, opsonization, and anaphylatoxins release) (71). Further, studies showed enhanced anti-tumor activity of mAbs by overpowering the effect of CRPs (67, 134). This has been proposed by researchers where a biotin-avidin system or bispecific monoclonal antibodies are used in order to target a tumor antigen and simultaneously block a CRP, in order to limit inhibitory factors in the TME (135–138).

As previously mentioned, CRC cancer cells may resist CDC through the decay accelerator “CD55” overexpression under hypoxic conditions. Hence, a study by Dho et al., 2019, explored the potential of a novel CD55 chimeric monoclonal antibody that suppressed proliferation, invasion and migration of CRC cells, through activating the complement system. Further, a synergistic action of the anti-CD55 antibody and 5-fluoruracil (5FU) was observed on CRC cells growth rate (139). The therapeutic potential of the complement system as an anti-cancer agent was translated in the clinical practice, where a phase I trial (STELLAR-001) has been designed to investigate IPH5401 (anti-C5aR) in combination with durvalumab (anti-PD-1) in advanced solid tumors (NCT03665129, https://clinicaltrials.gov/ct2/show/NCT03665129) (140).

Other factors including factor H were demonstrated to inactivate therapeutic ADCC. Antibodies targeting factor H were previously utilized in lung cancer studies to increase C3b deposition and mediate complement-dependent tumor cell lysis (141). Therefore, targeting factor H in cancer may be a potential strategy to overcome immune evasion and enhance tumor response to immunotherapy. Another strategy to boost the complement-mediated cytolysis is through the blockage of the MAC blocker “CD59”, such as that reported in lung cancer using trastuzumab and cetuximab (anti-EGFR) antibodies (123). In addition, another regulatory protein, CD46 (MPC) is a cofactor for C3b and C4b degradation by factor H, which represents another target for the cancer treatment (18).

## Conclusions

CRC is still considered among the most prevalent malignancies worldwide. The known treatment strategies to treat CRC patients are surgery and chemotherapy. Nevertheless, the prognosis of CRC has never been satisfying, especially for patients with metastatic lesions. As a crucial member of humoral innate immunity, the complement system was found to be present in the TME of various cancers. Recent research has shown that the complement can be pro or anti-tumoral, depending on the cancer type, and different investigated models. A deeper knowledge of the complement system’s interaction within TME will lead to a new breakthrough in cancer immunotherapy. Therefore, complement components and regulators represent a potential target for CRC immunotherapy.

## Author Contributions

IMT and NME proposed the outline of the review. IMT, NME, and MS wrote the first draft. NE prepared the figures. IMT, NME, and MS discussed and edited the text. All authors have read and agreed to the published version of the manuscript.

## Funding

This research was funded by MBRU-Al-Mahmeed Research Award 2019 (ALM1914).

## Conflict of Interest

The authors declare that the research was conducted in the absence of any commercial or financial relationships that could be construed as a potential conflict of interest.

## Publisher’s Note

All claims expressed in this article are solely those of the authors and do not necessarily represent those of their affiliated organizations, or those of the publisher, the editors and the reviewers. Any product that may be evaluated in this article, or claim that may be made by its manufacturer, is not guaranteed or endorsed by the publisher.
